# BMAL1 controls glucose uptake through paired-homeodomain transcription factor 4 in differentiated Caco-2 cells

**DOI:** 10.1152/ajpcell.00058.2019

**Published:** 2019-06-19

**Authors:** Whitney Sussman, Matthew Stevenson, Cyrus Mowdawalla, Samantha Mota, Louis Ragolia, Xiaoyue Pan

**Affiliations:** ^1^Department of Foundations of Medicine, New York University Long Island School of Medicine, Mineola, New York; ^2^Diabetes and Obesity Research Center, New York University Winthrop Hospital, Mineola, New York; ^3^Department of Cell Biology, State University of New York Downstate Medical Center, Brooklyn, New York

**Keywords:** BMAL1, Caco-2, PAX4, SGLT1, transporter

## Abstract

The transcription factor aryl hydrocarbon receptor nuclear translocator-like protein-1 (BMAL1) is an essential regulator of the circadian clock, which controls the 24-h cycle of physiological processes such as nutrient absorption. To examine the role of BMAL1 in small intestinal glucose absorption, we used differentiated human colon adenocarcinoma cells (Caco-2 cells). Here, we show that BMAL1 regulates glucose uptake in differentiated Caco-2 cells and that this process is dependent on the glucose transporter sodium-glucose cotransporter 1 (SGLT1). Mechanistic studies show that BMAL1 regulates glucose uptake by controlling the transcription of SGLT1 involving paired-homeodomain transcription factor 4 (PAX4), a transcriptional repressor. This is supported by the observation that clustered regularly interspaced short palindromic repeats (CRISPR)-CRISPR-associated endonuclease Cas9 (Cas9) knockdown of PAX4 increases SGLT1 and glucose uptake. Chromatin immunoprecipitation (ChIP) and ChIP-quantitative PCR assays show that the knockdown or overexpression of BMAL1 decreases or increases the binding of PAX4 to the hepatocyte nuclear factor 1-α binding site of the SGLT1 promoter, respectively. These findings identify BMAL1 as a critical mediator of small intestine carbohydrate absorption and SGLT1.

## INTRODUCTION

Circadian rhythms are ~24-h cycles of physiologic processes, allowing organisms to synchronize with their environment (16, 51, 52). The circadian feedback loop is located in various tissues throughout the body known as peripheral clocks, which are entrained by the suprachiasmatic nucleus as well as various other factors including nutrients, hormones, and body temperature (1, 8, 14, 21, 44). Studies in mice have suggested that the liver, pancreas, skeletal muscle, kidneys, and small intestine all have peripheral circadian clocks (9, 17–20, 30, 39, 45, 55). As evidenced by studies with the small intestine, not only can a tissue itself express clock genes, but also numerous biological processes can exhibit circadian rhythmicity, including DNA synthesis, motility, glucose transporters, and macronutrient absorption (3, 6, 11–13). Studies have shown that disruption to core clock components and/or clock-controlled genes results in numerous gastrointestinal abnormalities (19–22, 32, 45, 49, 53). Cellular uptake of glucose is mediated by sodium-glucose cotransporter 1 (SGLT1). SGLT1 was localized at the brush border membranes of intestinal epithelial cells (15, 26, 28, 56). We recently found that mice carrying a dominant-negative circadian locomotor output cycles protein kaput (CLOCK) mutant protein express elevated levels of mRNAs encoding glucose transporters such as glucose transporter 2 (GLUT2, a solute carrier family 2 member), GLUT5, and SGLT1. These glucose transporters exemplify the circadian clock’s critical role in glucose metabolism and imply a connection between a malfunctioning clock and increased intestinal glucose uptake (32). Other studies with mice have implicated that the clock gene, aryl hydrocarbon receptor nuclear translocator-like protein-1 (BMAL1), is pivotal for normal glucose uptake by the small intestine (2). Mice with a disruption to BMAL1 exhibit impaired glucose tolerance and decreased insulin secretion, resulting in hyperglycemia and a higher risk for type 2 diabetes mellitus compared with wild-type mice (27). Consequently, these BMAL1-mutant mice have significantly higher glucose levels and secrete less insulin throughout the day (27, 43). SGLT1 is an essential transporter of both sodium and glucose across the apical membrane of epithelial cells, and its primary function is to regulate glucose uptake by the intestine and kidney (15, 42). An SGLT1^−/−^ mouse model demonstrated that the absence of SGLT1 results in the complete ablation of sodium-dependent glucose cotransport across the brush border membrane in the small intestine in vivo (15). A link has been made between BMAL1 and SGLT1 through observation of protein-DNA interactions in vivo using a chromatin immunoprecipitation (ChIP) assay (23). Finally, BMAL1 binds tightly to the transcription initiation site of mouse SGLT1, suggesting that BMAL1 regulates the expression of this hexose transporter (23). It remains unclear whether BMAL1 binding actually promotes SGLT1 transcription, and this has not yet been confirmed in vitro using the human Caco-2 cell line.

Mutations in the enhancer box (E-box) and hepatocyte nuclear factor 1-α (HNF1α) binding sites of paired-homeodomain transcription factor 4 (PAX4) decrease the binding of PAX4 to its target promoters, which requires the action of activin A (24, 46, 47). PAX4, a transcriptional repressor, interacts with and represses the activity of transcription factors involved in glucose intolerance, hormone-directed homeostatic processes, and type 2 diabetes (46, 47). PAX4 is required for the development of the endocrine system, i.e., the epithelial invagination of the pancreas, a process beginning in utero with the development of the duodenum that continues into adulthood (46). Furthermore, PAX4 is involved in the differentiation of insulin-producing β- and δ-cells of the pancreas, and certain mutations in PAX4 can lead to the development of severe diabetes mellitus (46). PAX4-deficient mice exhibit a severe deficiency of gastrointestinal endocrine cell types. Recently, research has shown that the knockout of PAX4 upregulates ghrelin expression in the cells of the duodenum and pancreas in mice and development of hyperglycemia in rabbits (54, 57). In addition, PAX4 expression is guided by a diurnal rhythm in the mammalian pineal gland (40, 41). However, the function of PAX4 in the in vitro-differentiated human colon carcinoma cell line (differentiated Caco-2 cells) has not been thoroughly investigated. BMAL1 regulates some genes involved in lipid metabolism as well as the efflux of cholesterol from the liver into bile through transcription factors such as GATA-binding protein-4 (GATA4) and small heterodimer partner (SHP), which are encoded by clock-controlled genes (30, 33, 38). Therefore, circadian clock genes are likely regulating clock-controlled genes to modulate cellular physiology in other tissues.

Here, we show that the BMAL1-PAX4 axis plays a major role in normal glucose uptake and that BMAL1 deficiency or overexpression forces substantial cellular adaptations to the consequent functions of that axis. Using both loss- and gain-of-function approaches in Caco-2 cells, we show that BMAL1 regulates SGLT1 gene expression and glucose uptake in a PAX4-dependent manner.

## MATERIALS AND METHODS

### 

#### Materials.

2-NBD glucose (2-[*N*-(7-nitrobenz-2-oxa-1,3-diazol-4-yl)amino]-2-deoxyglucose) (cat. no. N-13195) was obtained from Invitrogen. [^14^C]methyl-α-d-[U-^14^C]glucopyranoside (NEC-659250-UC, [^14^C]αMG, 9.66 GBq/mmol) and [^3^H]mannitol (NET-10125-UC) were obtained from PerkinElmer. Rabbit polyclonal antibodies were purchased from different companies and used for Western blot analysis: anti-glyceraldehyde-3-phosphate dehydrogenase (GAPDH, ab-8245, diluted 1:1,000; Abcam), anti-SGLT1 (sc-98974, 1:1,000; Santa Cruz Biotechnology), and horseradish peroxidase-conjugated secondary antibodies (anti-rabbit IgG, cat. no. 31460; anti-mouse IgG, cat. no. 31430; 1:3,000; Invitrogen). Human BMAL1 siRNA (siBMAL1, smart pool, cat. no. 11865), siSHP (smart pool, cat. no. 003410), caudal-type homeobox protein-2 (CDX2) siRNA (siCDX2, smart pool, cat. no. 015636), siGATA4 (smart pool, cat. no. 014463), forkhead box protein O2 (FOXO2) siRNA (siFOXO2, smart pool, cat. no. 003007), D-box-binding proline- and acid-rich basic region leucine zipper (PAR bZIP) transcription factor (DBP) siRNA (siDBP, smart pool, cat. no. 017689), and siRNA control (cat. no. D-001206-13-05) were obtained from Dharmacon. BMAL1 shRNA lentiviral particles (sc-38165-v) and control shRNA lentiviral particles (sc-108080) were from Santa Cruz Biotechnology. Adenovirus (Adv)-BMAL1 (cat. no. 1445) and Adv-Control (cat. no. 1300) were from Vector Biolabs. The single-guide RNA (sgRNA)/clustered regularly interspaced short palindromic repeats (CRISPR)-associated endonuclease Cas9 (CAS9) all-in-one expression clone targeting PAX4 (cat. no. HCP212211-CG01-1-10) as well as scrambled-sequence sgRNA (cat. no. CCPCTR01-CG01-10) control for pCRISPR-CG01 were from GeneCopoeia, and anti-PAX4 (ab-101721, 1:100) and anti-Bmal1 (ab-93806, 1:100) were from Abcam (30, 33, 38).

#### Cells.

Caco-2 cells were obtained from the American Type Culture Collection (Rockville, MD) and were cultured (75-cm^2^ flasks; Corning Glassworks, Corning, NY) in Dulbecco’s modified Eagle’s medium (DMEM) containing high glucose level supplemented with 1% l-glutamine and 1% antibiotic-antimycotic mixture and 20% fetal bovine serum (FBS) in a humidified incubator at 37°C. For all experiments, Caco-2 cells from 70–80% confluent flasks were grown on 0.4-µm polycarbonate micropore membrane inserts (cat. no. 3414, Transwells, 6-well plate, 24-mm diameter; Corning Costar, Cambridge, MA) at a density of 1 × 10^5^ cells/cm^2^. To induce cell differentiation, the media were changed every other day for 21 days as described (32).

Differentiated Caco-2 cells were transduced with control shRNA [shControl, 1.2 × 10^6^ lentiviral transducing units (TU)/ml], shBMAL1 (1.2 × 10^6^ TU/ml), Adv-Control (1.2 × 10^6^ TU/ml), or Adv-BMAL1 (1.2 × 10^6^ TU/ml) for 72 h with a versatile and high-efficiency magnetofection magnetic transfection kit (cat. no. am-70000 and RL-40000; Boca Scientific) as described (29–31).

Differentiated Caco-2 cells were transfected with different siRNAs with silenceMag siRNA delivery reagent (cat. no. sm-10000; Boca Scientific) plus Lipofectamine 2000 (cat. no. 11668-019; Invitrogen; 10) for 72 h. Caco-2 cells were seeded 21 days for differentiation before transfection in six-well plates. To determine the optimal transfection conditions, 5 ng of each siRNA (20 μM stock) and siRNA control were transfected using the following ratios (wt/vol) of siRNA to transfection reagent. Lipofectamine 2000 (1:5) plus silenceMag siRNA delivery reagent (1:1.25) mixed in serum-free transfection medium (cat. no. sc-36868; Santa Cruz Biotechnology) was mixed according to the siRNA transfection protocol. Mixtures were incubated for 20 min at room temperature with a total volume of 2.5 ml in Transwells and mixed by pipetting. Mixtures were incubated for 20 min at room temperature and then mixed directly with 0% FBS DMEM cells for 12 h. The transfection mixtures then were removed, and 2 ml of 1X normal growth medium DMEM (containing 10% FBS) were added to each well. Cultivation was continued until 72 h after magnetofection, when cells were harvested to analyze for RNA or protein assay. Then, we collected the Caco-2 cells from the polycarbonate micropore membrane for protein or RNA measurement. In a separate experiment, Caco-2 cells were kept in a humidified incubator at 37°C and transduced with a scrambled control, pCRISPR-Control, specific for sgRNA/CAS9-PAX4 with or without shBMAL1. All cell samples were washed with 1 ml 1X phosphate-buffered saline (PBS) and centrifuged (433 *g*, 5 min) before being stored at −80°C until further experimentation was completed as described in previous studies (31, 35).

#### 2-NBD glucose uptake.

Differentiated Caco-2 cells were grown in DMEM containing 10% FBS in six-well cluster plates, and the medium was changed every 3 days for a total of 3 wk. Cells were kept in a humidified incubator at 37°C, and the cells were transduced with different viral particles. After 72 h of treatment, cells were treated with 25 µM 2-NBD glucose for 4 h in the humidified incubator, and then the cells were washed with PBS three times and photographed under a fluorescence confocal microscope. The amount of NBD glucose taken up by the cells was measured by direct fluorometry (485/535 nM) using a plate reader. The amount of fluorescence was normalized with protein for each group, and the shControl, and shBMAL1, Adv-Control, and Adv-BMAL1 groups were compared as previously described (33).

#### [^14^C]αMG uptake.

For glucose uptake experiments, differentiated Caco-2 cells were incubated with 1.0 μCi/ml [^14^C]αMG in 1 ml DMEM at 37°C at appointed time points. After washing with DMEM, the cells were washed with 1X PBS and extracted with 1 N NaOH for protein assay, and the total radioactivity of the cells was counted in a scintillation counter and normalized for protein as described in the literature (32, 36, 37).

#### Western blot studies.

The cell samples were stored overnight at −80°C, after which the protein was isolated from the cells using a buffer containing 10 mM Tris·HCl, 250 mM sucrose, 1 mM ethylenediaminetetracetic acid, and 0.5 mM phenylmethylsulfonyl fluoride (pH 7.5) as previously described (33–35). The extracted proteins were resolved on 10% SDS-polyacrylamide gels and were then electrophoretically transferred to a nitrocellulose membrane (Bio-Rad, Hercules, CA). Each membrane was blocked in Tris-buffered saline (TBS) buffer containing 0.2% Tween 20 (TBS-T) and 5% nonfat dry milk overnight at 4°C. Each membrane was incubated with a primary antibody against SGLT1 (1:1,000) or GAPDH (1:1,000) in TBS-T buffer for 2 h at 25°C, and then each membrane was incubated with a secondary antibody against rabbit (1:3,000) or against mouse (1:3,000) IgG, diluted in TBS-T buffer, for 2 h at 25°C. Immunopositive protein bands were scanned using a Bio-Rad densitometer, and the band intensity was analyzed and quantified using National Institutes of Health (NIH) imaging software as previously described (32, 35, 36).

#### Real-time PCR.

Real-time PCR was performed as described in the literature (32, 35–37, 58). Briefly, total RNA was isolated from the cells using TRIzol (Invitrogen), and the concentration of the RNA was assessed using a NanoDrop 2000 spectrophotometer. Then 1 µg RNA was subjected to reverse transcription to yield cDNA using the Omniscript RT kit (cat. no. 205113; Qiagen; 10-SN10-05; Eurogentec) in an Applied Biosystems QuantStudio 3 to evaluate the effect of different PCR primers (see Table 1) on the measurement of cellular mRNA levels by the 2^∆∆Ct^ method (where Ct is threshold cycle) and normalized to 18s rRNA, as described in previous studies (32, 35, 37, 58).

**Table 1.** Oligonucleotide sequences of primers used

	RT-PCR Primers	
	Forward (5′–3′)	Reverse (5′–3′)
hPAX4 promoter	GGTGATTTCAGGACCTCGGG	TCCTCAAACAGTCTAGCGCG
hSGLT1 promoter	CAGGACAGCTCTTACCTGCC	GCAAAGAGGGAGGCTCCAAT
hSGLT1 promoter for E-box	GCACCCATTTTACAAGCGGA	CACAAAGGCCCACTGTACTC
hSGLT1 promoter for HNF4α	GTCATGCCTCCTCTCTTGGT	GCTATTTCCTTGCCCTGTCC
hSGLT1 promoter for Sp1	GAGTACAGTGGGCCTTTGTG	GAGGGAGCAGGGCAGTAG
hSGLT1 promoter for HNF1α	TACTGCCCTGCTCCCTCAAA	TCCTTCCCTCTCGCCAGG
hHNF1α	CAGCCCACCTACGTTTAATCATT	CCAACTCCAAAATCAGGACCTA
hHNF1β	GGCAATTGCACAAATGTCCTCT	GGAGTCCTTGACATCGTGGG
hCDX2	CTCGGCAGCCAAGTGAAAAC	CAGAGAGCCCCAGCGTG
hSGLT1	TGGTGCACAGATCAGGTCAT	GGACGACACAGGCAATTTTT
hPAX4	TCCTTACAGCCCATTCCATC	CACCCTCATGCAGCTGTCTA
hPAX6	AGTGAATCAGCTCGGTGGTG	GTCTGATGGAGCCAGTCTCG
hSP1	TCATCCGGACACCAACAGTG	ATTGGAAATCTTACCTGGGGCA
hSHP	GCTTAGCCCCAAGGAATATGC	TTGGAGGCCTGGCACATC
hDBP	CCAGTTCCACGTATAGCTGAA	CAGCATATGTAGGTCACATGA
hFOXO1	AACCTGGCATTACAGTTGGCC	AAATGCAGGAGGCATGACTACGT
hFOXO2	GCCATGCACTCGGCTTCCAGTAT	CAGCGCCCACGTACGACGAC
hGATA4	TCCCTCTTCCCTCCTCAAAT	GTCCCATCAGCGTGTAAAGG
hGATA6	GCCTTGCCTGCTATGGAATA	ACCTCATGAACCGACTCAGC
hGAPDH	GAAAGCCTGCCGGTGACTAA	TTCCCGTTCTCAGCCTTGAC

CDX2, caudal-type homeobox protein-2; DBP, D-box-binding proline- and acid-rich basic region leucine zipper (PAR bZIP) transcription factor; E-box, enhancer box; FOXO1 and FOXO2, forkhead box proteins O1 and O2, respectively; GATA4 and GATA6, GATA-binding proteins-4 and -6, respectively; h, human; HNF1α, HNF1β, and HNF4α, hepatocyte nuclear factors 1-α, 1-β, and 4-α, respectively; PAX4 and PAX6, paired-homeodomain transcription factors 4 and 6, respectively; SGLT1, sodium-glucose cotransporter 1; SHP, small heterodimer partner; SP1, Sp1 transcription factor.

#### ChIP assay.

ChIP was used to study the binding of different transcription factors to the SGLT1 promoter using goat polyclonal antibodies against PAX4 or BMAL1 as previously described (29–31). The DNA samples recovered after immunoprecipitation were subjected to semiquantitative PCR or quantitative PCR to detect coimmunoprecipitated DNA using the SGLT1 promoter-specific primers in Table 1 that flank the consensus E-box binding site in the human SGLT1 promoter. As a negative control, ChIP was performed in the presence of normal rabbit IgG. As a positive control, ChIP was performed using input DNA. These experiments were performed three times with similar results. Data from a representative experiment are provided (30, 34, 38).

#### Statistical analyses.

Microsoft Excel and Microsoft PowerPoint programs were used to analyze the data. Data are presented as means ± SE. Statistical analysis was performed with the unpaired Student’s *t*-test. For comparison between two groups, a one-way ANOVA was performed followed by Dunnett’s correction. All statistical analyses were conducted using GraphPad Prism, and differences were considered significant at different levels, depending on the type of analysis: *P* < 0.05, *P* < 0.01, and *P* < 0.001.

## RESULTS

### 

#### BMAL1 regulates the uptake of glucose by differentiated Caco-2 cells.

**Fig. 1.**
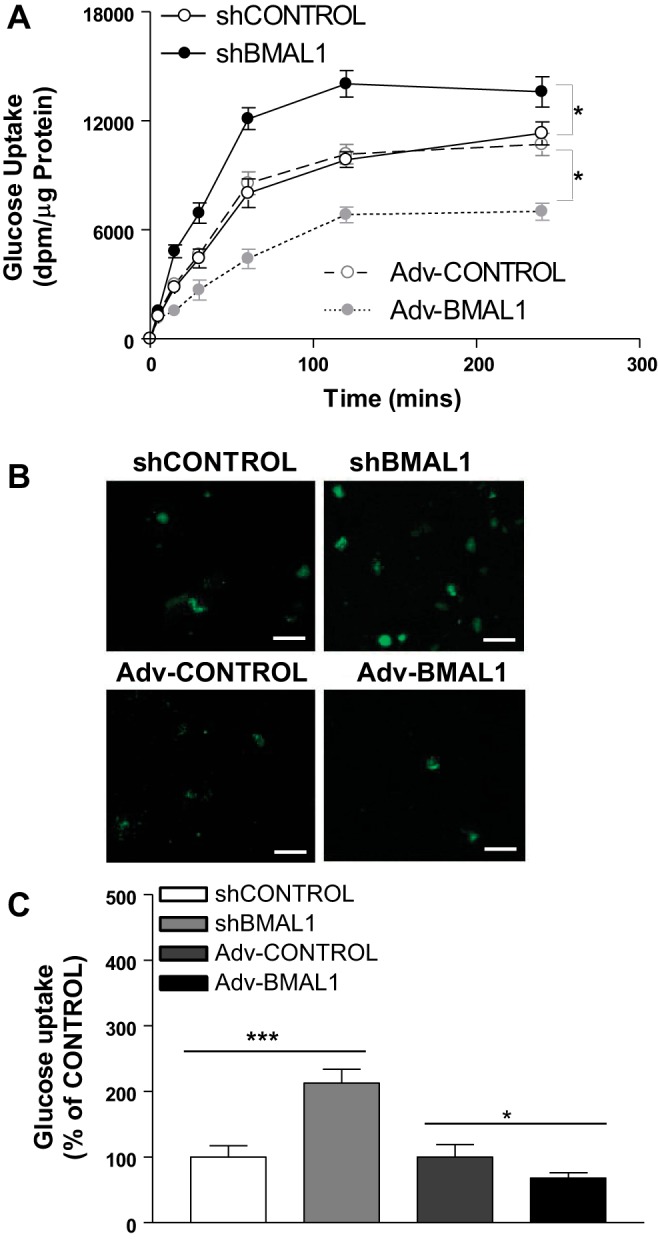
Effect of aryl hydrocarbon receptor nuclear translocator-like protein-1 (BMAL1) on glucose uptake. *A*: differentiated Caco-2 cells were transduced with control shRNA (shControl) [1.2 × 10^6^ lentiviral transducing units (TU)/ml], BMAL1 shRNA (shBMAL1, 1.2 × 10^6^ TU/ml), adenovirus (Adv)-Control (1.2 × 10^6^ TU/ml), or Adv-BMAL1 (1.2 × 10^6^ TU/ml) for 72 h. The accumulation and the transepithelial transport of [^14^C]methyl-α-d-[U-^14^C]glucopyranoside (1 µCi/ml) were determined at the indicated times. Each bar represents the mean ± SE of 3 separate trials. *P < 0.05, significantly different from shControl or Adv-Control, respectively. *B* and *C*: differentiated Caco-2 cells were transduced with shControl, shBMAL1, Adv-Control, or Adv-BMAL1 for 72 h and then treated with 2-NBD glucose (2-[*N*-(7-nitrobenz-2-oxa-1,3-diazol-4-yl)amino]-2-deoxyglucose) (25 mM) for 4 h, and then Caco-2 cells were photographed under a fluorescence confocal microscope. Scale bars: 100 µm (*B*). We then collected and measured the amount of 2-NBD glucose taken up by direct fluorometry (485/535 nM) using a plate reader; the amount of fluorometry was normalized with protein for each group (*C*). We repeated this experiment 3 times, and we calculated the mean of these 3 measurements. Each group represents the mean ± SE of 3 measurements. *P < 0.05, ***P < 0.001, significantly different from each Control group.

Previous studies with mice revealed that the circadian clock genes regulate glucose absorption in the small intestine (4, 32). To understand how BMAL1 regulates differentiated Caco-2 cells’ glucose uptake, we first compared glucose uptake between cells expressing a control shRNA (shControl) and Adv-Control, which revealed no difference in glucose uptake (Fig. 1*A*). The knockdown of BMAL1 with a specific shRNA (shBMAL1) significantly increased the amount of [^14^C]αMG taken up by cells compared with the shControl groups (Fig. 1*A*). However, the overexpression of BMAL1 (Adv-BMAL1) significantly decreased the amount of [^14^C]αMG in cells compared with the Adv-Control groups (Fig. 1*A*). Cells from the various treatment groups were observed under a fluorescence microscope to determine whether BMAL1 directly regulates glucose uptake using a fluorescent glucose (2-NBD glucose; Fig. 1, *B* and *C*). Cells treated with Adv-BMAL1 had significantly less fluorescence intensity than either shBMAL1- or Adv-Control-treated cells (*P* < 0.05; Fig. 1*B*). The increase in fluorescence observed for shBMAL1-treated cells reflects an increase in glucose uptake, whereas the decrease in fluorescence for the Adv-BMAL1-treated cells indicates a reduction in glucose uptake (Fig. 1*C*). These results suggest that BMAL1 regulates glucose uptake in Caco-2 cells.

#### BMAL1 regulates the expression of the gene SGLT1 in differentiated Caco-2 cells.

**Fig. 2.**
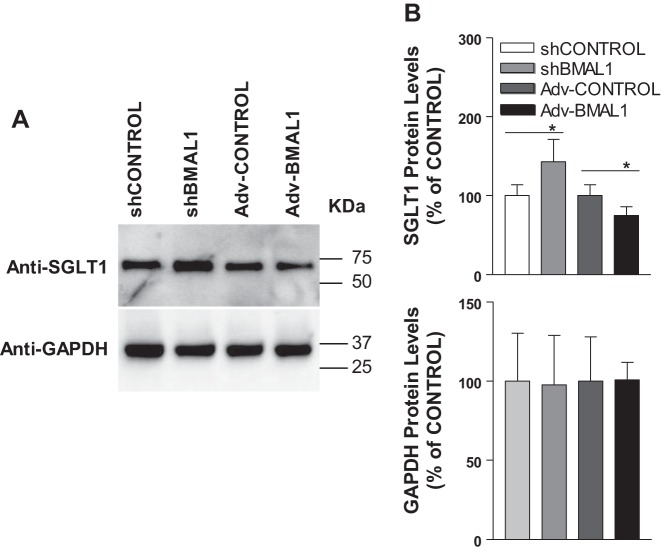
Effect of aryl hydrocarbon receptor nuclear translocator-like protein-1 (BMAL1) on sodium-glucose cotransporter 1 (SGLT1) protein level in Caco-2 cells. *A*: differentiated Caco-2 cells were transduced with control shRNA (shControl), BMAL1 shRNA (shBMAL1), adenovirus (Adv)-Control, or Adv-BMAL1 for 72 h. Cells were collected, and soluble lysate was prepared. *B*: total proteins (20 µg per lane) of cell lysates were subjected to Western blot analysis with the indicated antibodies, and protein abundance is expressed as a percentage of shControl and Adv-Control, respectively. The data were quantified, and each bar represents the mean ± SE of 3 independent trials. *P < 0.05, significantly different from each Control.

To understand the mechanisms by which BMAL1 regulates cellular glucose uptake, we analyzed the correlation between BMAL1 and SGLT1 levels with Western blot using differentiated Caco-2 cells transfected with shControl, or shBMAL1, Adv-Control, or Adv-BMAL1 (Fig. 2). The intensity of these bands was analyzed and quantified using NIH imaging software (Fig. 2*A*). As a housekeeping gene, the cellular level of the GAPDH protein expression remained relatively constant among all cell groups (Fig. 2*A*). Caco-2 cells with decreased BMAL1 expression contained ~50% more SGLT1 protein compared with the shControl group (Fig. 2*B*). An increase in BMAL1 expression, however, resulted in a 25% reduction in SGLT1 level compared with the Adv-Control group (Fig. 2*B*). These respective increases and decreases in SGLT1 protein level and activity confirm that BMAL1 regulates glucose uptake via the regulation of SGLT1 in vitro.

**Fig. 3.**
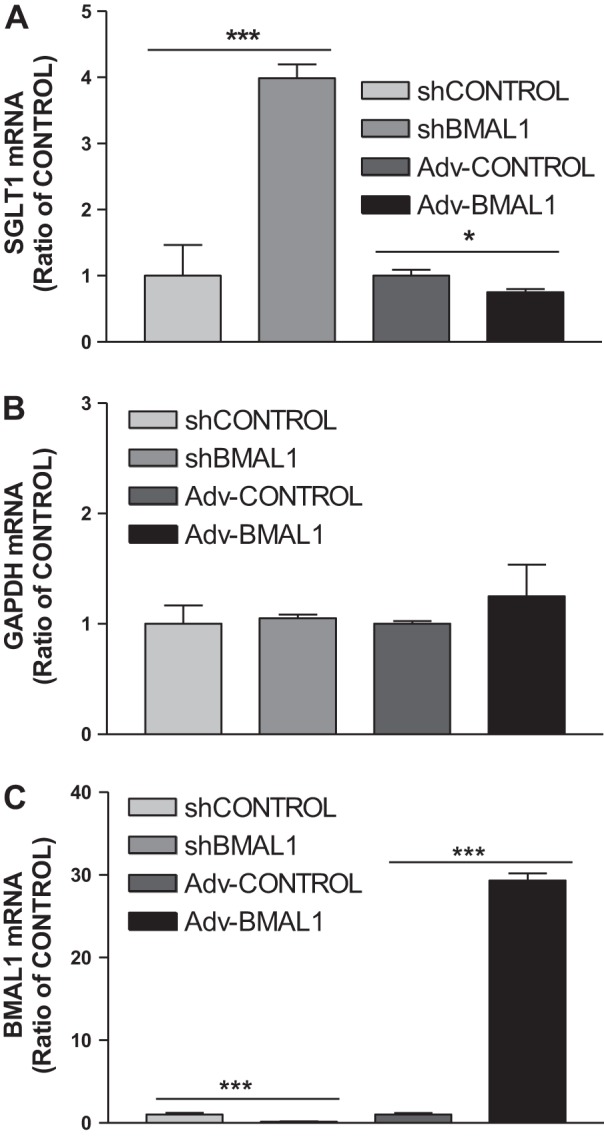
Effect of aryl hydrocarbon receptor nuclear translocator-like protein-1 (BMAL1) on sodium-glucose cotransporter 1 (SGLT1) mRNA expression level. Differentiated Caco-2 cells were transduced with control shRNA (shControl), BMAL1 shRNA (shBMAL1), adenovirus (Adv)-Control, or Adv-BMAL1 for 72 h, and cells were used to quantify mRNA levels of SGLT1 (*A*), GAPDH (*B*), and BMAL1 (*C*). Each bar represents the mean ± SE of 3 separate trials. *P < 0.05, ***P < 0.001, significantly different from each Control.

These results suggest that BMAL1 is a transcription factor that regulates both SGLT1 level and SGLT1-mediated glucose uptake by Caco-2 cells. Therefore, we chose to explore the mechanism underlying BMAL1 transcriptional regulation of SGLT1 (Fig. 3). We used real-time PCR to assess SGLT1 mRNA level, which revealed a significant increase in SGLT1 mRNA level (*P* < 0.001) in shBMAL1-treated cells compared with shControl, and SGLT1 mRNA level decreased significantly (*P* < 0.05) in BMAL1-overexpressing cells compared with Adv-Control (Fig. 3*A*). GAPDH expression remained relatively constant in both treatment groups compared with the shControl and Adv-Control groups (Fig. 3*B*). Because an increase or decrease in BMAL1 expression decreased or increased SGLT1 mRNA level, respectively, BMAL1 seemed to have a direct impact on SGLT1 levels and hence on SGLT1 activity.

#### BMAL1 regulates the expression of the gene SGLT1 by modulating the transcription factor gene PAX4.

**Fig. 4.**
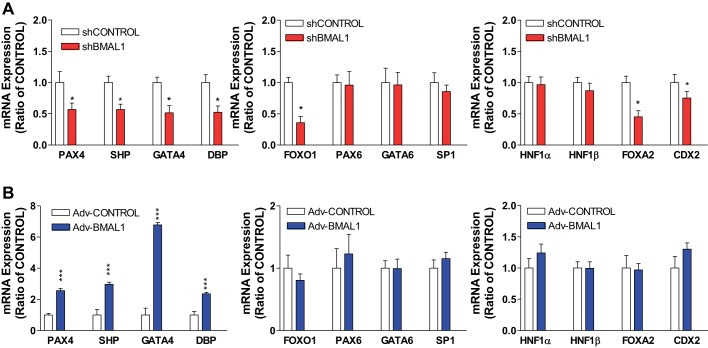
Effect of aryl hydrocarbon receptor nuclear translocator-like protein-1 (BMAL1) on mRNA expression levels of paired-homeodomain transcription factor 4 (PAX4) and different transcription factors. Differentiated Caco-2 cells were transduced with control shRNA (shControl) or BMAL1 shRNA (shBMAL1) (*A*) or adenovirus (Adv)-Control or Adv-BMAL1 (*B*) for 72 h and then used to quantify mRNA levels of PAX4 and different transcription factors. Each bar represents the mean ± SE of 3 separate trials. CDX2, caudal-type homeobox protein-2; DBP, D-box-binding proline- and acid-rich basic region leucine zipper (PAR bZIP) transcription factor; FOXA2 and FOXO1, forkhead box proteins A2 and O1, respectively; GATA4 and GATA6, GATA-binding proteins-4 and -6, respectively; HNF1α and HNF1β, hepatocyte nuclear factors 1-α and 1-β, respectively; SHP, small heterodimer partner; SP1, Sp1 transcription factor. *P < 0.05, ***P < 0.001, significantly different from each Control.

**Fig. 5.**
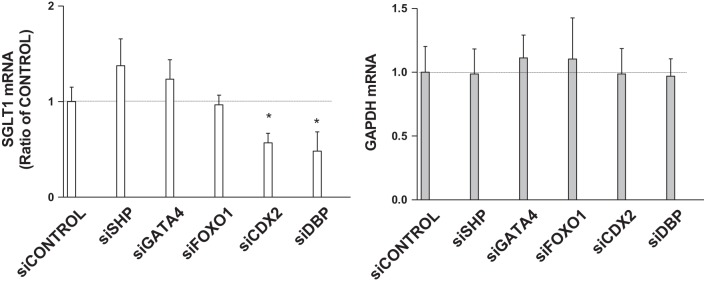
Expression of sodium-glucose cotransporter 1 (SGLT1) mRNA after siRNA-induced knockdown of different transcription factors. Differentiated Caco-2 cells were transfected with different siRNAs with Lipofectamine 2000 plus silenceMag siRNA delivery reagent for 72 h and then used to quantify mRNA levels of SGLT1 and GAPDH genes. Each bar represents the mean ± SE of 3 separate trials. CDX2, caudal-type homeobox protein-2; DBP, D-box-binding proline- and acid-rich basic region leucine zipper (PAR bZIP) transcription factor; FOXO1, forkhead box protein O1; GATA4, GATA-binding protein-4; SHP, small heterodimer partner; si, siRNA. *P < 0.05, significantly different from siControl.

**Fig. 6.**
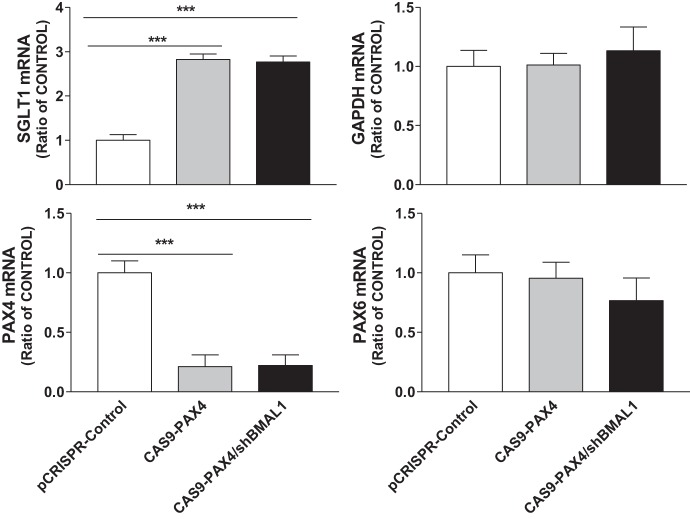
Effect of paired-homeodomain transcription factor 4 (PAX4) on the expression of sodium-glucose cotransporter 1 (SGLT1) in Caco-2 cells treated with clustered regularly interspaced short palindromic repeats plasmid (pCRISPR)-Control or target PAX4 with or without aryl hydrocarbon receptor nuclear translocator-like protein-1 shRNA (shBMAL1). Differentiated Caco-2 cells were transduced with a different scrambled control (pCRISPR-Control) or CRISPR-associated endonuclease Cas9 (CAS9)-PAX4 with or without shBMAL1 for 72 h. Cells were used to quantify mRNA levels of SGLT1 and different genes. Each bar represents the mean ± SE of 3 separate trials. ***P < 0.001, significantly different from scrambled control group.

We tested whether the cellular levels of these transcription factors respond to shBMAL1 or Adv-BMAL1 in differentiated Caco-2 cells to regulate SGLT1. In principle, increased expression of activators or decreased expression of the repressors in shBMAL1-treated cells might explain the observed increase in SGLT1 levels. Treatment of cells with shBMAL1 decreased the levels of DBP and CDX2 mRNA (Fig. 4*A*). However, the levels of two activators, namely, HNF1α and HNF1β, as well as Sp1 transcription factor (SP1) were not altered, and the level of another transcription factor, forkhead box protein A2 (FOXA2), was reduced (Fig. 4*A*). This suggests that the effect of BMAL1 on SGLT1 transcription might not involve these activators. Therefore, we studied changes in the expression of several repressor genes that are regulated by BMAL1. Treatment of cells with shBMAL1 reduced the levels of mRNAs encoding SHP, PAX4, and GATA4 but did not significantly affect the transcriptional activators PAX6 and GATA4 (Fig. 4*A*). This indicates that BMAL1 may enhance SGLT1 expression by suppressing the cellular levels of SHP and PAX4, but not PAX6. In turn, overexpression of BMAL1 increased PAX4, SHP, GATA4, and DBP expression but had no effect on FOXO1, FOXA2, SP1, or CDX2 (Fig. 4*B*). These data suggest that BMAL1 mainly regulates PAX4, SHP, GATA4, and DBP transcription factors. To understand how these clock-controlled genes regulate SGLT1 in Caco-2 cells, we knocked down the levels of various transcription factor mRNAs via the expression of specific siRNAs. Knockdown of SHP, GATA4, or FOXO1 did not affect SGLT1 expression, but knockdown of CDX2 or DBP decreased SGLT1 expression (Fig. 5). To determine whether PAX4 affects SGLT1 expression, Caco-2 cells were transfected with virus encoding Cas9-PAX4. This treatment significantly decreased PAX4 mRNA expression but increased SGLT1 mRNA expression, whereas the levels of PAX6 and GAPDH mRNAs were not affected (Fig. 6). Cotransfection with shBMAL1 and Cas9-PAX4 significantly increased SGLT1 expression, indicating that BMAL1 may regulate SGLT1 through the repressive function of PAX4.

**Fig. 7.**
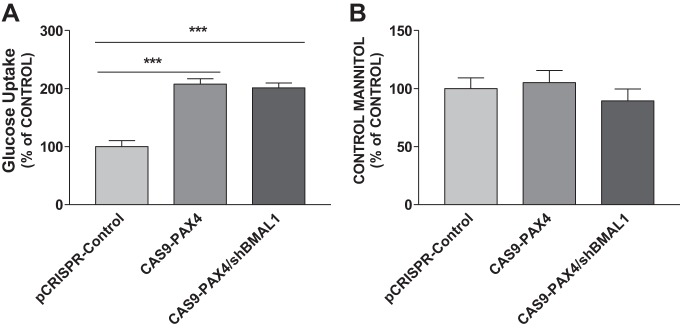
Effect of paired-homeodomain transcription factor 4 (PAX4) on glucose uptake in Caco-2 cells. Differentiated Caco-2 cells were transduced with a scrambled control [clustered regularly interspaced short palindromic repeats plasmid (pCRISPR)-Control] or CRISPR-associated endonuclease Cas9 (CAS9)-PAX4 with or without aryl hydrocarbon receptor nuclear translocator-like protein-1 shRNA (shBMAL1) for 72 h. The Caco-2 cells were then incubated in 1 µCi/ml of [^14^C]methyl-α-d-[U-^14^C]glucopyranoside ([^14^C]αMG) for 2 h. The accumulation and the transepithelial transport of [^14^C]αMG were determined (*A*). The [^3^H]mannitol radioactivity was measured simultaneously for validation of paracellular flux (*B*). Each bar represents the mean ± SE of 3 separate trials. ***P < 0.001, significantly different from scrambled control group.

To further investigate the functional relationship between PAX4 and SGLT1, we studied SGLT1’s role in glucose uptake using [^14^C]αMG. Decreased expression of PAX4 resulted in an increase in [^14^C]αMG uptake by differentiated Caco-2 cells (Fig. 7*A*); the uptake of mannitol (control) was not affected (Fig. 7*B*). Cotransfection with viruses encoding shBMAL1 and Cas9-PAX4 significantly increased glucose uptake (Fig. 7*A*). These studies indicate that decreased PAX4 expression activates glucose uptake by upregulating SGLT1 expression.

#### PAX4 indirectly binds to the promoter of SGLT1 through HNF1α.

**Fig. 8.**
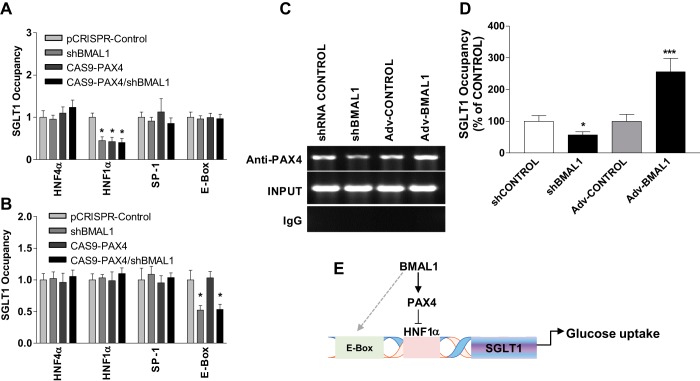
Binding of paired-homeodomain transcription factor 4 (PAX4) to *cis*-elements in the sodium-glucose cotransporter 1 (SGLT1) promoter. *A*–*D*: binding of PAX4 to different *cis*-elements in the SGLT1 promoter. Differentiated Caco-2 cells were transduced with clustered regularly interspaced short palindromic repeats plasmid (pCRISPR)-Control, single-guide RNA (sgRNA)/CRISPR-associated endonuclease Cas9 (CAS9)-PAX4, aryl hydrocarbon receptor nuclear translocator-like protein-1 shRNA (shBMAL1), or sgRNA/CAS9-PAX4/shBMAL1. After 72 h, the cells were subjected to a chromatin immunoprecipitation (ChIP) assay with anti-PAX4 (1:50, anti-PAX4 immunoprecipitated DNA) (*A*) and anti-BMAL1 (1:100, anti-BMAL11 immunoprecipitated DNA) (*B*), and immunoprecipitates were used to amplify sequences corresponding to hepatocyte nuclear factor 4-α (HNF4α), HNF1α, Sp1 transcription factor (SP1), and enhancer box (E-box) *cis*-elements. SGLT1 promoter sequences in the immunoprecipitates were subjected to quantitative PCR (*B*). Caco-2 cells were transduced with control shRNA (shControl), BMAL1 shRNA (shBMAL1), adenovirus (Adv)-Control, or Adv-BMAL1. After 72 h, the cells were subjected to ChIP using anti-PAX4 (1:50, anti-PAX4 immunoprecipitated DNA), anti-IgG (IgG immunoprecipitated DNA), and total DNA (INPUT) from differentiated Caco-2 cells after treatment with shBMAL1 or Adv-BMAL1, and then immunoprecipitates were used to amplify sequences corresponding to HNF1α in the SGLT1 promoter (*C*). SGLT1 promoter sequences in the immunoprecipitates from *C* were also subjected to quantitative PCR (*D*). Each bar represents the mean ± SE of 3 separate trials. *P < 0.05, ***P < 0.001, significantly different from scrambled control group. *E*: our data show that BMAL1 may regulate PAX4 to modulate SGLT1 and glucose uptake. BMAL1 positively regulates PAX4 (as a repressor), to inhibit the binding of HNF1α to SGLT1 promoter. Knockdown of BMAL1 decreases PAX4 expression and then induces SGLT1 to upregulate glucose uptake; in turn, overexpression of BMAL1 increases PAX4 expression, to inhibit the binding of HNF1α to SGLT1 promoter, and decreases SGLT1 expression to downregulate glucose uptake.

Attempts were then made to understand how PAX4 suppresses SGLT1 expression. Several groups have shown that different transcription factors regulate SGLT1 expression through binding to the SGLT1 promoter. Therefore, we studied the binding of PAX4 or BMAL1 to HNF1α and HNF4α in the SGLT1 promoter. We found that SP1 could bind its putative binding site within the SGLT1 promoter. Under normal conditions (i.e., Control), anti-PAX4-precipitated sequences were occupied in the SGLT1 promoter by HNF4α, HNF1α, Sp1, and E-box (Fig. 8*A*). The binding of PAX4 to the HNF1α binding site of the SGLT1 promoter was significantly reduced in shBMAL1-, Cas9-PAX4-, and Cas9-PAX4/shBMAL1-treated cells (Fig. 8*A*). The binding of PAX4 to the binding sites for E-box, HNF4α, or Sp-1 within the SGLT1 promoter was not affected in Cas9-PAX4-, shBMAL1-, or Cas9-PAX4/shBMAL1-treated cells. Compared with PAX4, the binding of BMAL1 to the E-box binding site of the SGLT1 promoter was significantly decreased in shBMAL1- and Cas9-PAX4/shBMAL1-treated cells, but this was not the case for Cas9-PAX4-treated cells (Fig. 8*B*). Finally, knockdown or overexpression of BMAL1 decreased or increased, respectively, the binding of PAX4 to the HNF1α binding site of the SGLT promoter (Fig. 8, *C* and *D*). These studies indicate that the knockdown of either BMAL1 or PAX4 significantly decreases the binding of PAX4 to the SGLT1 promoter.

## DISCUSSION

Our results provide a new understanding of the mechanisms involved in the uptake of glucose by intestinal cells. The data demonstrate that BMAL1 can act at the cellular level to modulate glucose uptake and SGLT1 expression in differentiated Caco-2 cells in a manner dependent on its role in PAX4-regulated transcription expression of SGLT1.

In this study, we observed that BMAL1 knockdown increased the level of SGLT1 mRNA in differentiated Caco-2 cells. Surprisingly, our studies reveal that PAX4 is required for the increased expression of SGLT1 observed in Caco-2 cells upon BMAL1 knockdown. Although ChIP assays using cells of the small intestine have established that clock genes can directly bind the SGLT1 promoter independent of E-box status (3), certain other functions of BMAL1 require PAX4 to regulate SGLT1 in vitro. Our study presents convincing evidence that BMAL1 contributes to cellular glucose uptake, which is consistent with previous reports (3, 5). Furthermore, we found that PAX4 represses the binding of HNF1α to the SGLT1 promoter (Fig. 8*E*). Our data show that BMAL1 positively regulates PAX4 (as a repressor), to inhibit the binding of HNF1α to SGLT1 promoter. Knockdown of BMAL1 decreases PAX4 expression and then induces SGLT1 to upregulate glucose uptake; in turn, overexpression of BMAL1 increases PAX4 expression, to inhibit the binding of HNF1α to SGLT1 promoter, and decreases SGLT1 expression to downregulate glucose uptake. We previously reported that BMAL1 regulates lipid metabolism in the liver, and this effect appears to be mediated, at least in part, by SHP and GATA4 (30, 38). Here, we demonstrate that BMAL1 is directly involved in glucose uptake in a manner dependent on PAX4 in differentiated Caco-2 cells. The rate of nutrient absorption in the small intestine varies during a 24-h period and is regulated by circadian clock genes (3, 6, 19, 20, 32, 45). We and other groups have shown that circadian clock genes are expressed in intestinal stem cells, colonic epithelial cells, and enterocytes (19, 20, 25, 29, 32, 45, 48). We previously demonstrated that mutations in certain circadian clock genes increase glucose uptake and glucose absorption by enterocytes and the small intestine (32). Here, we are the first to show that the transcription factor BMAL1 has an effect on glucose uptake as well as SGLT1 gene expression at the transcription level through regulating PAX4 in differentiated Caco-2 cells. However, we still do not know whether other clock genes also regulate PAX4 in the small intestine.

Although it is well established that PAX4 as a transcriptional repressor regulates glucose intolerance, hormone-directed homeostatic processes, and type 2 diabetes (46), this study is the first to demonstrate that PAX4 can also regulate glucose uptake. Therefore, PAX4 might be involved in the regulation of glucose uptake in intestine cells. To our knowledge, studies have not measured PAX4 physiological function in intestinal cells. PAX4 is essential for development of β-cells (54). Our results do not exclude the possibility that gut-pancreas signaling might regulate PAX4 and influence SGLT1 transcription.

Iwashina et al. showed that the SGLT1 promoter has several E-boxes (23). Therefore, modulating BMAL1 in cells should correspond to modulated SGLT1 level through the direct binding of BMAL1 to E-box to enhance SGLT1 expression (23). BMAL1 is a heterodimer transcription factor, and when bound to the CLOCK protein, can bind to DNA and activate target genes. However, our results demonstrate that BMAL1 has the opposite effect on SGLT1 gene expression: decreasing BMAL1 expression actually increases SGLT1 expression (Figs. 1–3), and increasing BMAL1 expression decreases SGLT1 expression (Figs. 1–3). A study has shown that knockdown of period circadian protein homolog 1 (Per1) increased native SGLT1 expression in Caco-2 cells independent of E-box (3). There are several studies that have shown that knockdown of BMAL1 decreases Per1 expression (50–52). Our findings are consistent with previous reports (3). These data suggest that BMAL1 might also regulate SGLT1 through Per1 in Caco-2 cells. It is notable that knockdown of BMAL1 not only selectively downregulated PAX4 to increase SGLT1 expression but also can abolish the SGLT1 rhythm through E-box, although more experiments are needed to measure SGLT1 cycle expression by serum shock (7, 33, 38).

A number of studies have identified several types of transcription factors that are involved in the transcription of SGLT1; these are SP1, DBP, CDX2, HNF1α, and HNF1β (26, 28). Our study extends the repertoire of BMAL1-mediated PAX4 gene activation by showing that BMAL1 can regulate the SGLT1 promoter within the HNF1α binding site. Our findings are consistent with previous studies demonstrating that HNF1α is required for PAX4 regulation (40, 41, 54). PAX4 represses SGLT1 via HNF1α. As we still do not understand how BMAL1 positively regulates PAX4, further experiments will be needed to determine its mechanism.

A major caveat of our present study is that we used only differentiated Caco-2 cells in culture. Although this limits our scope, it was intentional as we wanted to test the specific hypothesis that BMAL1 may exert cell-specific effects on gene expression that are independent of signaling related to bile acids, gut-brain neuronal connections, and gut hormones (55). Therefore, future in vivo studies are required to establish the physiological relevance of this cell culture model. Future studies involving glucose synthesis and glucose secretion into the portal vein in vivo after global or tissue-specific BMAL1 knockout in mice may also provide novel insights.

Moreover, our published data have initially shown that differentiated Caco-2 cells might express circadian rhythm after serum shock (7). It is possible that high or low BMAL1 expression might induce a phase shift in the cell’s clock after serum shock. So far, it seems likely that BMAL1 may contribute to glucose uptake and SGLT1 via PAX4 in differentiated Caco-2 cells under normal conditions. Our work, however, does not exclude the possibility that high or low BMAL1 expression might phase shift the Caco-2 cells’ clocks rather than upregulate or downregulate SGLT1 and glucose uptake.

In summary, our results suggest a novel mechanism by which BMAL1 promotes glucose uptake. We show that BMAL1 regulates PAX4 to control SGLT1 expression and glucose uptake. This provides evidence that PAX4 may act locally to modulate glucose uptake. This further emphasizes the importance of this regulatory pathway in carbohydrate/glucose absorption by the small intestine under different physiological conditions and circadian behavior such as wakening, daily activities, and sleeping.

## GRANTS

This work was supported in part by NIH National Heart, Lung, and Blood Institute Grant R56 HL137912-01 and American Heart Association Grant-In-Aid 16GRNT30960027 to X. Pan.

## DISCLOSURES

No conflicts of interest, financial or otherwise, are declared by the authors.

## AUTHOR CONTRIBUTIONS

X.P. conceived and designed research; W.S., M.S., C.M., S.M., and X.P. performed experiments; W.S., M.S., and X.P. analyzed data; W.S., M.S., C.M., L.R., and X.P. interpreted results of experiments; W.S. and X.P. prepared figures; X.P. edited and revised manuscript; X.P. drafted manuscript; W.S., M.S., C.M., S.M., L.R., and X.P. approved final version of manuscript.

## 
